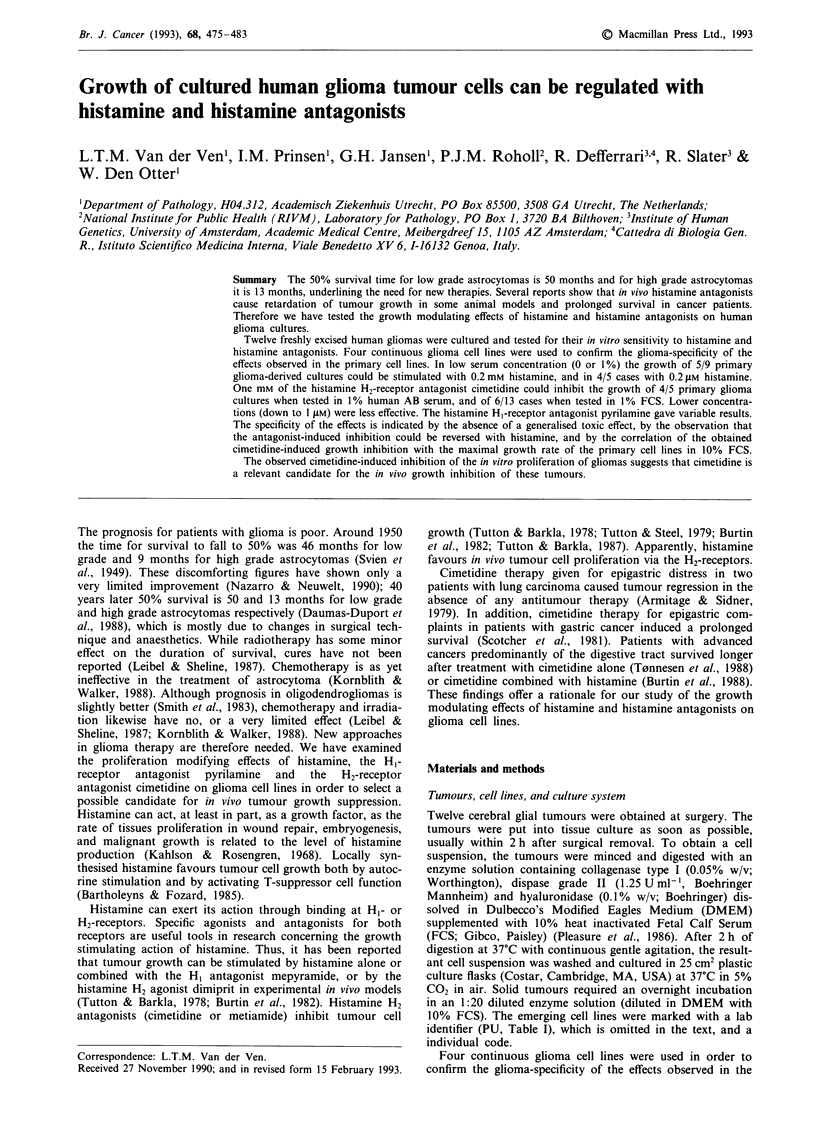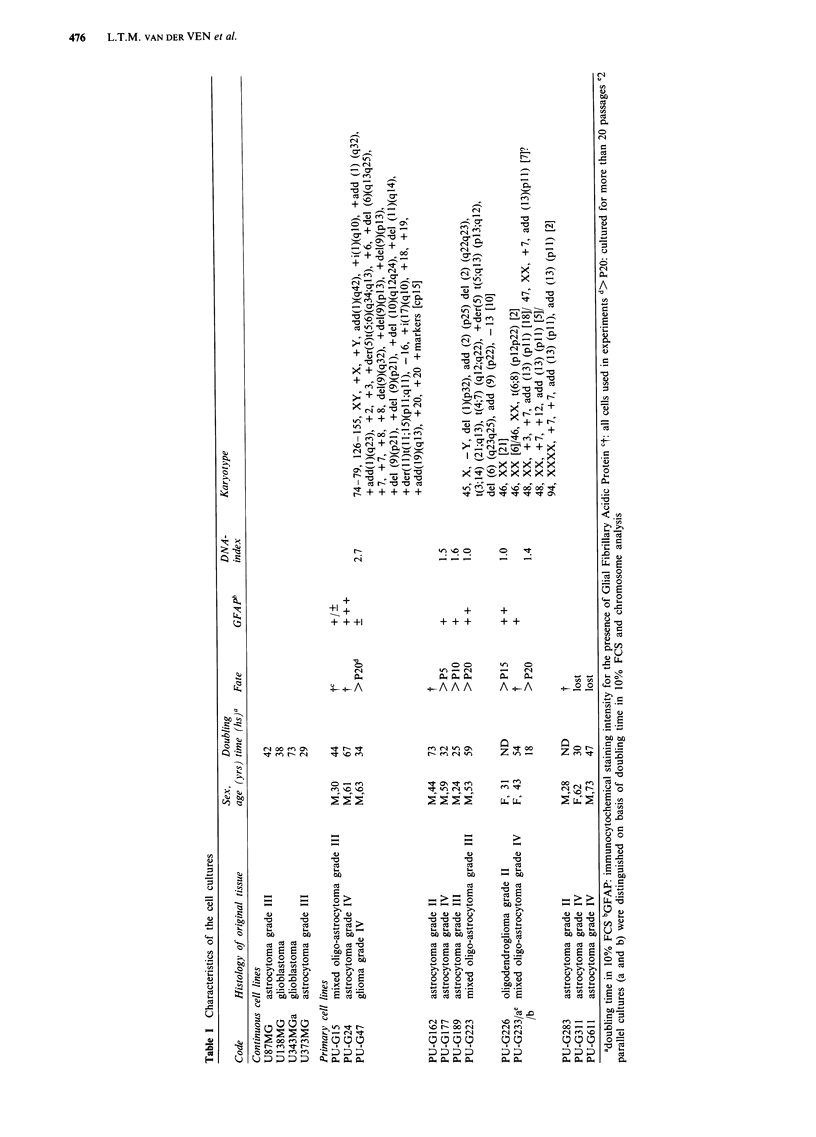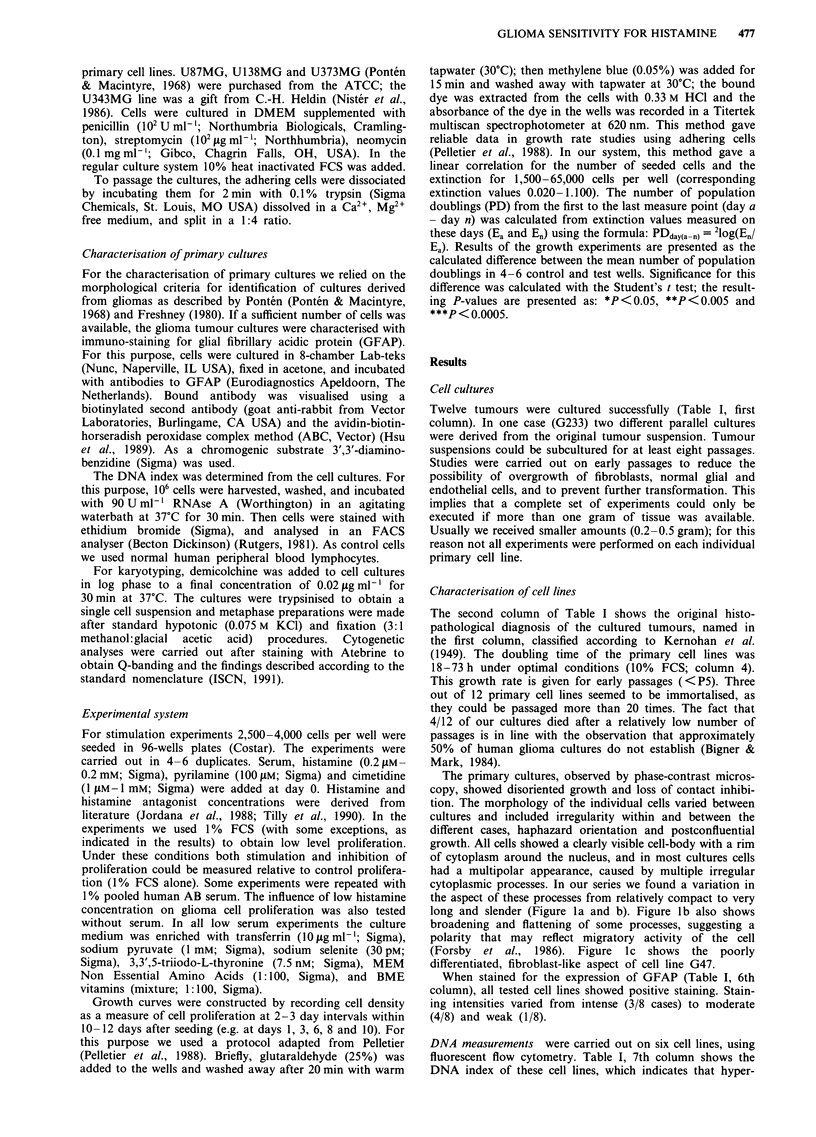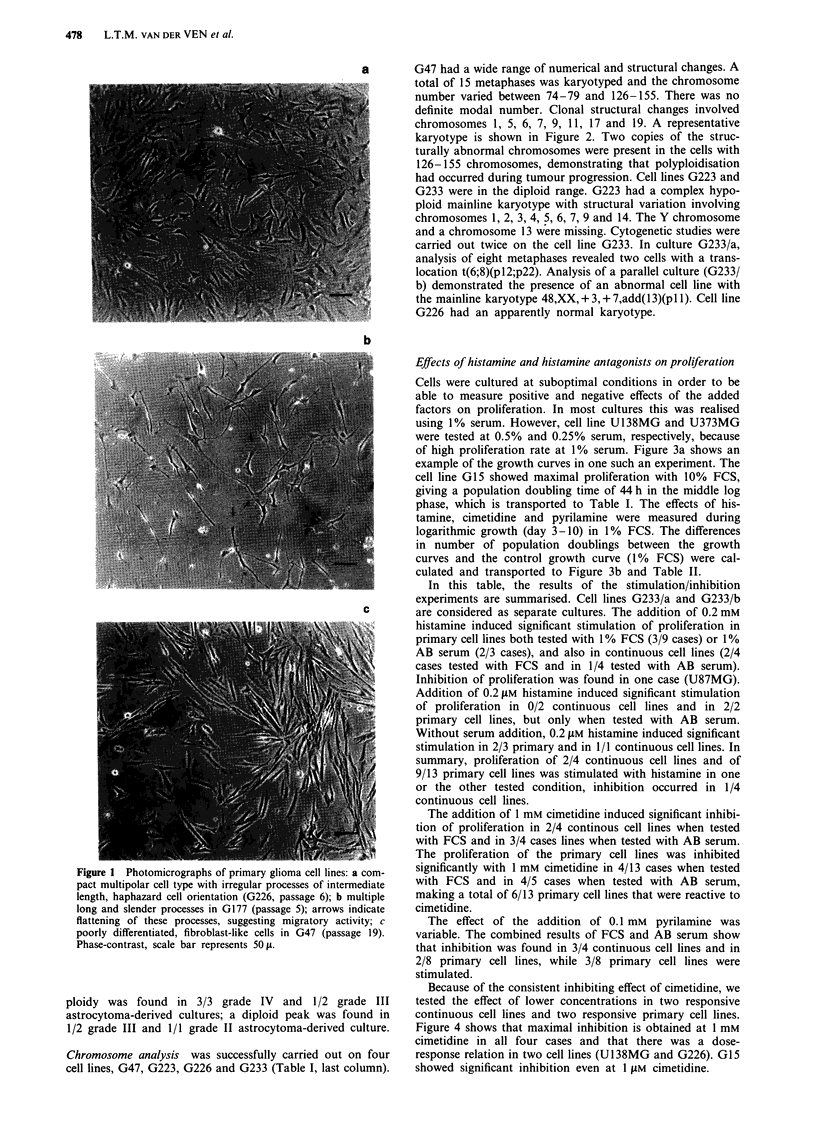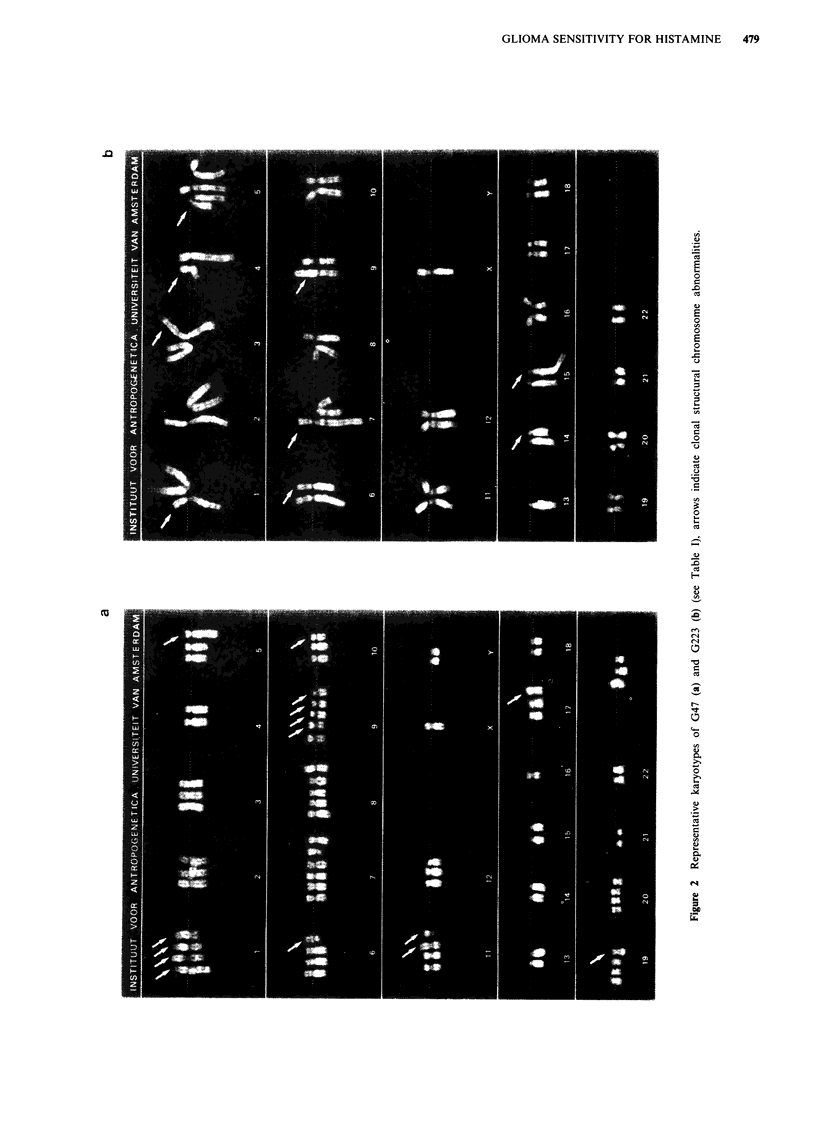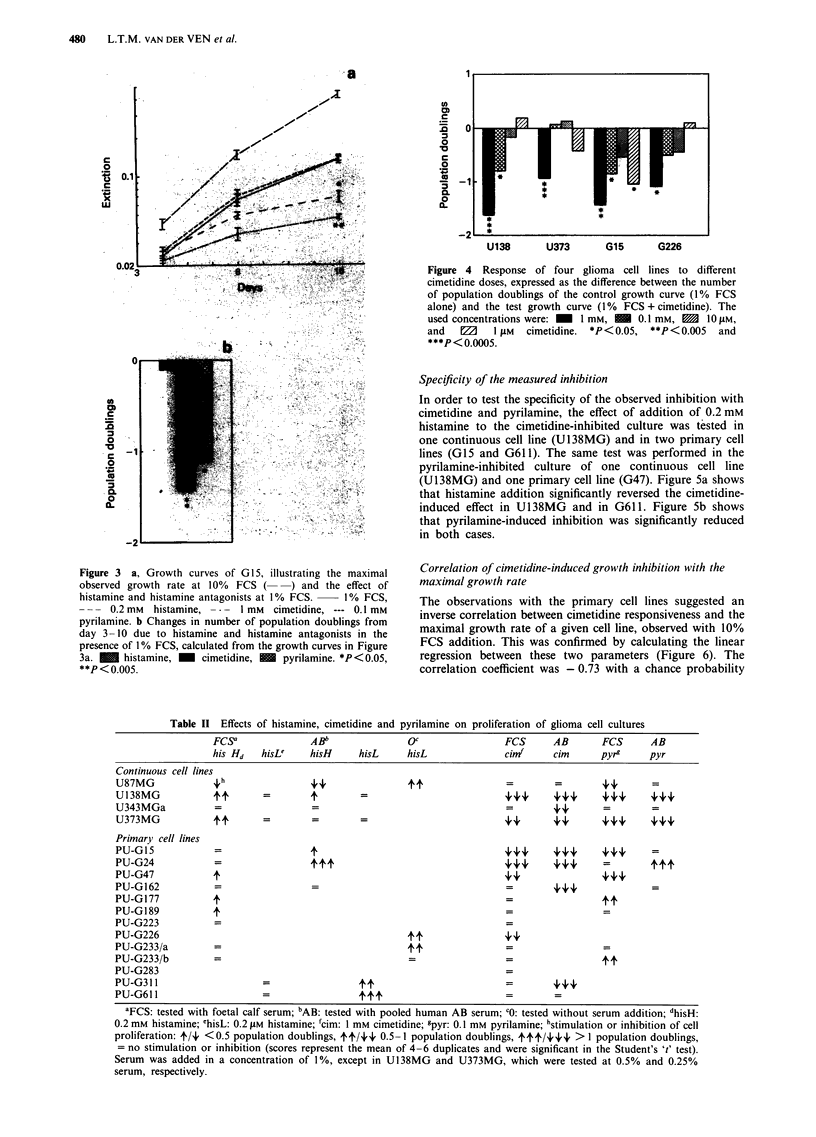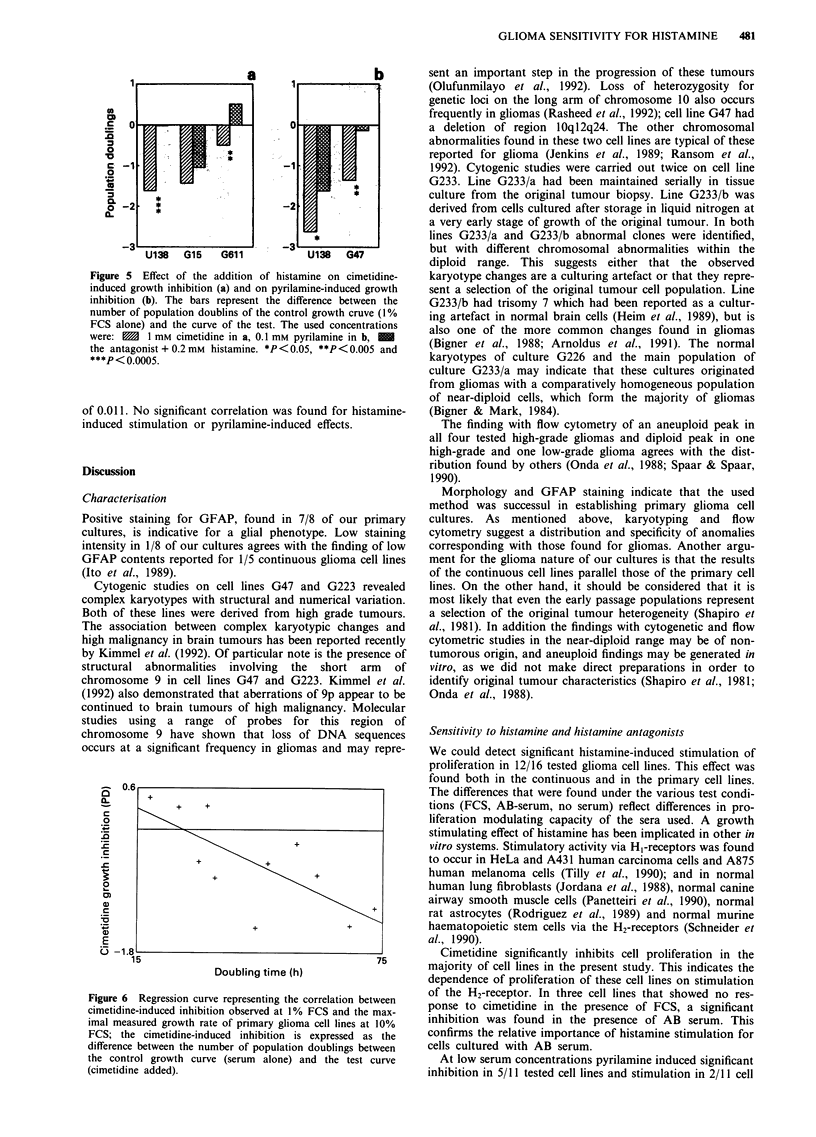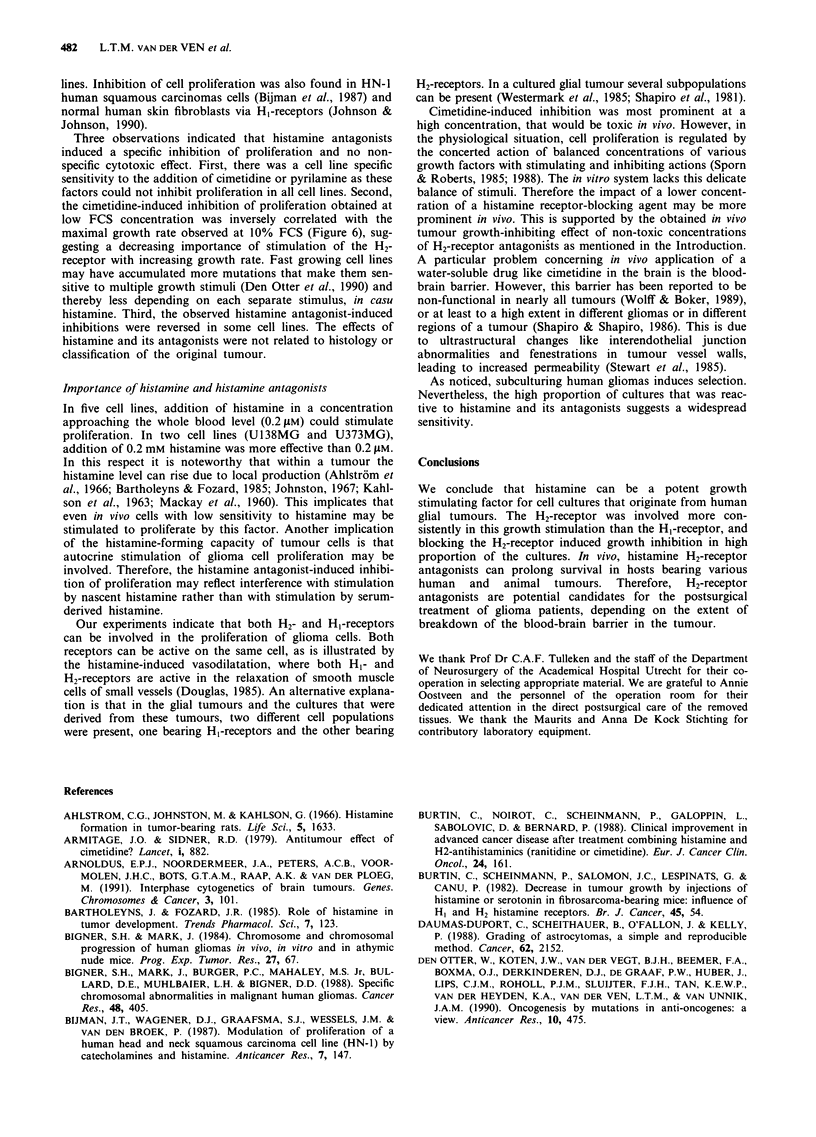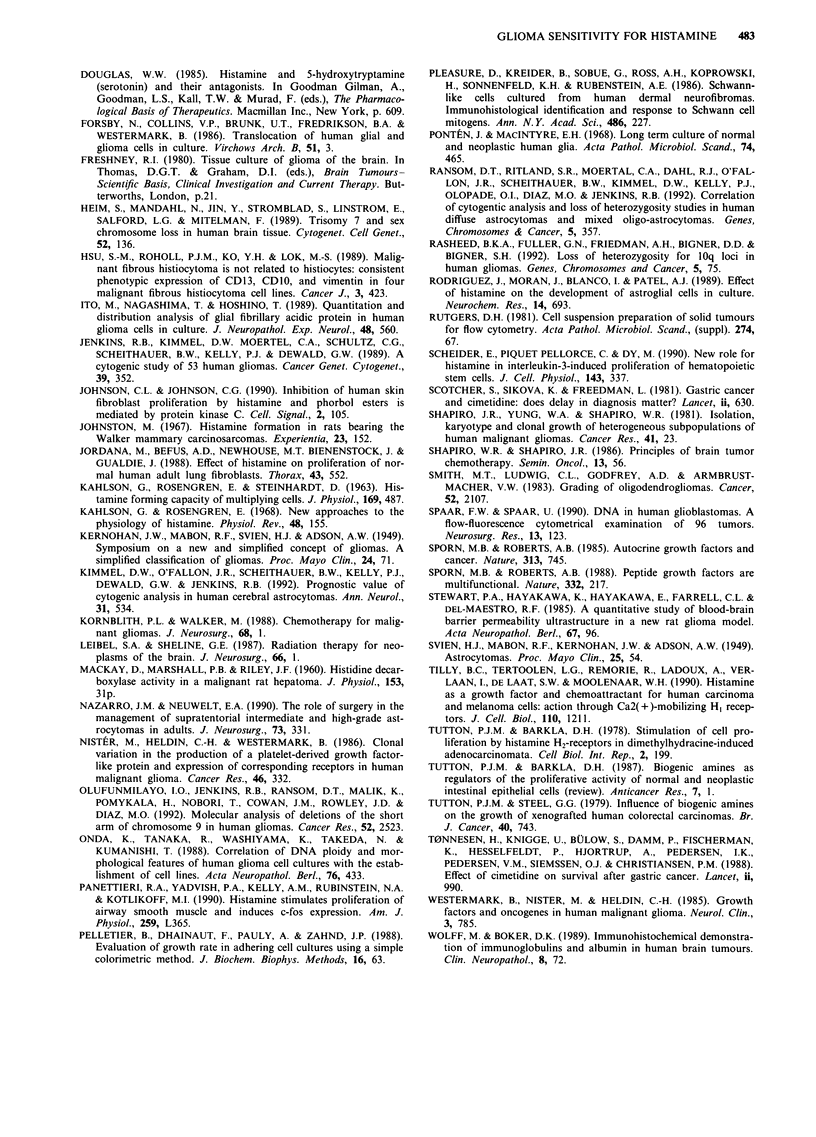# Growth of cultured human glioma tumour cells can be regulated with histamine and histamine antagonists.

**DOI:** 10.1038/bjc.1993.373

**Published:** 1993-09

**Authors:** L. T. Van der Ven, I. M. Prinsen, G. H. Jansen, P. J. Roholl, R. Defferrari, R. Slater, W. Den Otter

**Affiliations:** Department of Pathology, Academisch Ziekenhuis Utrecht, The Netherlands.

## Abstract

**Images:**


*Br. J. Cancer* (1993), **68**, 475–483 © Macmillan Press Ltd., 1993

### Growth of cultured human glioma tumour cells can be regulated with histamine and histamine antagonists

L.T.M. Van der Ven[1], I.M. Prinsen[1], G.H. Jansen[1], P.J.M. Roholl[2], R. Defferrari[3,4], R. Slater[3] & W. Den Otter[1]

[1]*Department of Pathology, H04.312, Academisch Ziekenhuis Utrecht, PO Box 85500, 3508 GA Utrecht, The Netherlands;* [2]*National Institute for Public Health (RIVM), Laboratory for Pathology, PO Box 1, 3720 BA Bilthoven;* [3]*Institute of Human Genetics, University of Amsterdam, Academic Medical Centre, Meibergdreef 15, 1105 AZ Amsterdam;* [4]*Cattedra di Biologia Gen. R., Istituto Scientifico Medicina Interna, Viale Benedetto XV 6, I-16132 Genoa, Italy.*

**Summary** The 50% survival time for low grade astrocytomas is 50 months and for high grade astrocytomas it is 13 months, underlining the need for new therapies. Several reports show that *in vivo* histamine antagonists cause retardation of tumour growth in some animal models and prolonged survival in cancer patients. Therefore we have tested the growth modulating effects of histamine and histamine antagonists on human glioma cultures.

Twelve freshly excised human gliomas were cultured and tested for their *in vitro* sensitivity to histamine and histamine antagonists. Four continuous glioma cell lines were used to confirm the glioma-specificity of the effects observed in the primary cell lines. In low serum concentration (0 or 1%) the growth of 5/9 primary glioma-derived cultures could be stimulated with 0.2 mM histamine, and in 4/5 cases with 0.2 $\mu$M histamine. One mM of the histamine $H_2$-receptor antagonist cimetidine could inhibit the growth of 4/5 primary glioma cultures when tested in 1% human AB serum, and of 6/13 cases when tested in 1% FCS. Lower concentrations (down to 1 $\mu$M) were less effective. The histamine $H_1$-receptor antagonist pyrilamine gave variable results. The specificity of the effects is indicated by the absence of a generalised toxic effect, by the observation that the antagonist-induced inhibition could be reversed with histamine, and by the correlation of the obtained cimetidine-induced growth inhibition with the maximal growth rate of the primary cell lines in 10% FCS.

The observed cimetidine-induced inhibition of the *in vitro* proliferation of gliomas suggests that cimetidine is a relevant candidate for the *in vivo* growth inhibition of these tumours.

The prognosis for patients with glioma is poor. Around 1950 the time for survival to fall to 50% was 46 months for low grade and 9 months for high grade astrocytomas (Svien *et al.*, 1949). These discomforting figures have shown only a very limited improvement (Nazarro & Neuwelt, 1990); 40 years later 50% survival is 50 and 13 months for low grade and high grade astrocytomas respectively (Daumas-Duport *et al.*, 1988), which is mostly due to changes in surgical technique and anaesthetics. While radiotherapy has some minor effect on the duration of survival, cures have not been reported (Leibel & Sheline, 1987). Chemotherapy is as yet ineffective in the treatment of astrocytoma (Kornblith & Walker, 1988). Although prognosis in oligodendrogliomas is slightly better (Smith *et al.*, 1983), chemotherapy and irradiation likewise have no, or a very limited effect (Leibel & Sheline, 1987; Kornblith & Walker, 1988). New approaches in glioma therapy are therefore needed. We have examined the proliferation modifying effects of histamine, the $H_1$-receptor antagonist pyrilamine and the $H_2$-receptor antagonist cimetidine on glioma cell lines in order to select a possible candidate for *in vivo* tumour growth suppression. Histamine can act, at least in part, as a growth factor, as the rate of tissues proliferation in wound repair, embryogenesis, and malignant growth is related to the level of histamine production (Kahlson & Rosengren, 1968). Locally synthesised histamine favours tumour cell growth both by autocrine stimulation and by activating T-suppressor cell function (Bartholeyns & Fozard, 1985).

Histamine can exert its action through binding at $H_1$- or $H_2$-receptors. Specific agonists and antagonists for both receptors are useful tools in research concerning the growth stimulating action of histamine. Thus, it has been reported that tumour growth can be stimulated by histamine alone or combined with the $H_1$ antagonist mepyramide, or by the histamine $H_2$ agonist dimiprit in experimental *in vivo* models (Tutton & Barkla, 1978; Burtin *et al.*, 1982). Histamine $H_2$ antagonists (cimetidine or metiamide) inhibit tumour cell growth (Tutton & Barkla, 1978; Tutton & Steel, 1979; Burtin *et al.*, 1982; Tutton & Barkla, 1987). Apparently, histamine favours *in vivo* tumour cell proliferation via the $H_2$-receptors.

Cimetidine therapy given for epigastric distress in two patients with lung carcinoma caused tumour regression in the absence of any antitumour therapy (Armitage & Sidner, 1979). In addition, cimetidine therapy for epigastric complaints in patients with gastric cancer induced a prolonged survival (Scotcher *et al.*, 1981). Patients with advanced cancers predominantly of the digestive tract survived longer after treatment with cimetidine alone (Tønnesen *et al.*, 1988) or cimetidine combined with histamine (Burtin *et al.*, 1988). These findings offer a rationale for our study of the growth modulating effects of histamine and histamine antagonists on glioma cell lines.

### Materials and methods

### *Tumours, cell lines, and culture system*

Twelve cerebral glial tumours were obtained at surgery. The tumours were put into tissue culture as soon as possible, usually within 2 h after surgical removal. To obtain a cell suspension, the tumours were minced and digested with an enzyme solution containing collagenase type I (0.05% w/v; Worthington), dispase grade II (1.25 U ml$^{-1}$, Boehringer Mannheim) and hyaluronidase (0.1% w/v; Boehringer) dissolved in Dulbecco's Modified Eagles Medium (DMEM) supplemented with 10% heat inactivated Fetal Calf Serum (FCS; Gibco, Paisley) (Pleasure *et al.*, 1986). After 2 h of digestion at 37°C with continuous gentle agitation, the resultant cell suspension was washed and cultured in 25 cm$^2$ plastic culture flasks (Costar, Cambridge, MA, USA) at 37°C in 5% $CO_2$ in air. Solid tumours required an overnight incubation in an 1:20 diluted enzyme solution (diluted in DMEM with 10% FCS). The emerging cell lines were marked with a lab identifier (PU, Table I), which is omitted in the text, and a individual code.

Four continuous glioma cell lines were used in order to confirm the glioma-specificity of the effects observed in the

Correspondence: L.T.M. Van der Ven.
Received 27 November 1990; and in revised form 15 February 1993.

**Table I** Characteristics of the cell cultures

| Code | Histology of original tissue | Sex, age (yrs) | Doubling time (hs)[a] | Fate | GFAP[b] | DNA-index | Karyotype |
|---|---|---|---|---|---|---|---|
| *Continuous cell lines* | | | | | | | |
| U87MG | astrocytoma grade III | | 42 | | | | |
| U138MG | glioblastoma | | 38 | | | | |
| U343MGa | glioblastoma | | 73 | | | | |
| U373MG | astrocytoma grade III | | 29 | | | | |
| *Primary cell lines* | | | | | | | |
| PU-G15 | mixed oligo-astrocytoma grade III | M,30 | 44 | †[c] | +/± | | |
| PU-G24 | astrocytoma grade IV | M,61 | 67 | † | +++ | | |
| PU-G47 | glioma grade IV | M,63 | 34 | >P20[d] | ± | 2.7 | 74–79, 126–155, XY, +X, +Y, add(1)(q42), +i(1)(q10), +add (1) (q32), +add(1)(q23), +2, +3, +der(5)t(5;6)(q34;q13), +6, +del (6)(q13q25), +7, +7, +8, +8, del(9)(q32), +del(9)(p13), +del(9)(p13), +del (9)(p21), +del (9)(p21), +del (10)(q12q24), +del (11)(q14), +der(11)t(11;15)(p11;q11), −16, +i(17)(q10), +18, +19, +add(19)(q13), +20, +20 +markers [cp15] |
| PU-G162 | astrocytoma grade II | M,44 | 73 | † | | | |
| PU-G177 | astrocytoma grade IV | M,59 | 32 | >P5 | + | 1.5 | |
| PU-G189 | astrocytoma grade III | M,24 | 25 | >P10 | + | 1.6 | |
| PU-G223 | mixed oligo-astrocytoma grade III | M,53 | 59 | >P20 | ++ | 1.0 | 45, X, −Y, del (1)(p32), add (2) (p25) del (2) (q22q23), t(3;14) (21;q13), t(4;7) (q12;q22), +der(5) t(5;q13) (p13;q12), del (6) (q23q25), add (9) (p22), −13 [10] |
| PU-G226 | oligodendroglioma grade II | F, 31 | ND | >P15 | ++ | 1.0 | 46, XX [21] |
| PU-G233/a[e] | mixed oligo-astrocytoma grade IV | F, 43 | 54 | † | + | | 46, XX [6]/46, XX, t(6;8) (p12p22) [2] |
| /b | | | 18 | >P20 | | 1.4 | 48, XX, +3, +7, add (13) (p11) [18]/ 47, XX, +7, add (13)(p11) [7]?<br>48, XX, +7, +12, add (13) (p11) [5]/<br>94, XXXX, +7, +7, add (13) (p11), add (13) (p11) [2] |
| PU-G283 | astrocytoma grade II | M,28 | ND | † | | | |
| PU-G311 | astrocytoma grade IV | F,62 | 30 | lost | | | |
| PU-G611 | astrocytoma grade IV | M,73 | 47 | lost | | | |

[a]doubling time in 10% FCS [b]GFAP: immunocytochemical staining intensity for the presence of Glial Fibrillary Acidic Protein [c]†: all cells used in experiments [d]>P20: cultured for more than 20 passages [e]2 parallel cultures (a and b) were distinguished on basis of doubling time in 10% FCS and chromosome analysis primary cell lines. U87MG, U138MG and U373MG (Pontén & Macintyre, 1968) were purchased from the ATCC; the U343MG line was a gift from C.-H. Heldin (Nistér *et al.*, 1986). Cells were cultured in DMEM supplemented with penicillin ($10^2$ U ml$^{-1}$; Northumbria Biologicals, Cramlington), streptomycin ($10^2$ μg ml$^{-1}$; Northhumbria), neomycin (0.1 mg ml$^{-1}$; Gibco, Chagrin Falls, OH, USA). In the regular culture system 10% heat inactivated FCS was added.

To passage the cultures, the adhering cells were dissociated by incubating them for 2 min with 0.1% trypsin (Sigma Chemicals, St. Louis, MO USA) dissolved in a $Ca^{2+}$, $Mg^{2+}$ free medium, and split in a 1:4 ratio.

*Characterisation of primary cultures*

For the characterisation of primary cultures we relied on the morphological criteria for identification of cultures derived from gliomas as described by Pontén (Pontén & Macintyre, 1968) and Freshney (1980). If a sufficient number of cells was available, the glioma tumour cultures were characterised with immuno-staining for glial fibrillary acidic protein (GFAP). For this purpose, cells were cultured in 8-chamber Lab-teks (Nunc, Naperville, IL USA), fixed in acetone, and incubated with antibodies to GFAP (Eurodiagnostics Apeldoorn, The Netherlands). Bound antibody was visualised using a biotinylated second antibody (goat anti-rabbit from Vector Laboratories, Burlingame, CA USA) and the avidin-biotin-horseradish peroxidase complex method (ABC, Vector) (Hsu *et al.*, 1989). As a chromogenic substrate 3′,3′-diamino-benzidine (Sigma) was used.

The DNA index was determined from the cell cultures. For this purpose, $10^6$ cells were harvested, washed, and incubated with 90 U ml$^{-1}$ RNAse A (Worthington) in an agitating waterbath at 37°C for 30 min. Then cells were stained with ethidium bromide (Sigma), and analysed in an FACS analyser (Becton Dickinson) (Rutgers, 1981). As control cells we used normal human peripheral blood lymphocytes.

For karyotyping, demicolchine was added to cell cultures in log phase to a final concentration of 0.02 μg ml$^{-1}$ for 30 min at 37°C. The cultures were trypsinised to obtain a single cell suspension and metaphase preparations were made after standard hypotonic (0.075 M KCl) and fixation (3:1 methanol:glacial acetic acid) procedures. Cytogenetic analyses were carried out after staining with Atebrine to obtain Q-banding and the findings described according to the standard nomenclature (ISCN, 1991).

*Experimental system*

For stimulation experiments 2,500–4,000 cells per well were seeded in 96-wells plates (Costar). The experiments were carried out in 4–6 duplicates. Serum, histamine (0.2 μM–0.2 mM; Sigma), pyrilamine (100 μM; Sigma) and cimetidine (1 μM–1 mM; Sigma) were added at day 0. Histamine and histamine antagonist concentrations were derived from literature (Jordana *et al.*, 1988; Tilly *et al.*, 1990). In the experiments we used 1% FCS (with some exceptions, as indicated in the results) to obtain low level proliferation. Under these conditions both stimulation and inhibition of proliferation could be measured relative to control proliferation (1% FCS alone). Some experiments were repeated with 1% pooled human AB serum. The influence of low histamine concentration on glioma cell proliferation was also tested without serum. In all low serum experiments the culture medium was enriched with transferrin (10 μg ml$^{-1}$; Sigma), sodium pyruvate (1 mM; Sigma), sodium selenite (30 pM; Sigma), 3,3′,5-triiodo-L-thyronine (7.5 nM; Sigma), MEM Non Essential Amino Acids (1:100, Sigma), and BME vitamins (mixture; 1:100, Sigma).

Growth curves were constructed by recording cell density as a measure of cell proliferation at 2–3 day intervals within 10–12 days after seeding (e.g. at days 1, 3, 6, 8 and 10). For this purpose we used a protocol adapted from Pelletier (Pelletier *et al.*, 1988). Briefly, glutaraldehyde (25%) was added to the wells and washed away after 20 min with warm tapwater (30°C); then methylene blue (0.05%) was added for 15 min and washed away with tapwater at 30°C; the bound dye was extracted from the cells with 0.33 M HCl and the absorbance of the dye in the wells was recorded in a Titertek multiscan spectrophotometer at 620 nm. This method gave reliable data in growth rate studies using adhering cells (Pelletier *et al.*, 1988). In our system, this method gave a linear correlation for the number of seeded cells and the extinction for 1,500–65,000 cells per well (corresponding extinction values 0.020–1.100). The number of population doublings (PD) from the first to the last measure point (day *a* – day *n*) was calculated from extinction values measured on these days ($E_a$ and $E_n$) using the formula: $PD_{day(a-n)} = {}^2\log(E_n/E_a)$. Results of the growth experiments are presented as the calculated difference between the mean number of population doublings in 4–6 control and test wells. Significance for this difference was calculated with the Student's *t* test; the resulting *P*-values are presented as: $^{*}P<0.05$, $^{**}P<0.005$ and $^{***}P<0.0005$.

### Results

*Cell cultures*

Twelve tumours were cultured successfully (Table I, first column). In one case (G233) two different parallel cultures were derived from the original tumour suspension. Tumour suspensions could be subcultured for at least eight passages. Studies were carried out on early passages to reduce the possibility of overgrowth of fibroblasts, normal glial and endothelial cells, and to prevent further transformation. This implies that a complete set of experiments could only be executed if more than one gram of tissue was available. Usually we received smaller amounts (0.2–0.5 gram); for this reason not all experiments were performed on each individual primary cell line.

*Characterisation of cell lines*

The second column of Table I shows the original histo-pathological diagnosis of the cultured tumours, named in the first column, classified according to Kernohan *et al.* (1949). The doubling time of the primary cell lines was 18–73 h under optimal conditions (10% FCS; column 4). This growth rate is given for early passages (<P5). Three out of 12 primary cell lines seemed to be immortalised, as they could be passaged more than 20 times. The fact that 4/12 of our cultures died after a relatively low number of passages is in line with the observation that approximately 50% of human glioma cultures do not establish (Bigner & Mark, 1984).

The primary cultures, observed by phase-contrast microscopy, showed disoriented growth and loss of contact inhibition. The morphology of the individual cells varied between cultures and included irregularity within and between the different cases, haphazard orientation and postconfluential growth. All cells showed a clearly visible cell-body with a rim of cytoplasm around the nucleus, and in most cultures cells had a multipolar appearance, caused by multiple irregular cytoplasmic processes. In our series we found a variation in the aspect of these processes from relatively compact to very long and slender (Figure 1a and b). Figure 1b also shows broadening and flattening of some processes, suggesting a polarity that may reflect migratory activity of the cell (Forsby *et al.*, 1986). Figure 1c shows the poorly differentiated, fibroblast-like aspect of cell line G47.

When stained for the expression of GFAP (Table I, 6th column), all tested cell lines showed positive staining. Staining intensities varied from intense (3/8 cases) to moderate (4/8) and weak (1/8).

*DNA measurements* were carried out on six cell lines, using fluorescent flow cytometry. Table I, 7th column shows the DNA index of these cell lines, which indicates that hyper-

**Figure 1** Photomicrographs of primary glioma cell lines: **a** compact multipolar cell type with irregular processes of intermediate length, haphazard cell orientation (G226, passage 6); **b** multiple long and slender processes in G177 (passage 5); arrows indicate flattening of these processes, suggesting migratory activity; *c* poorly differentiated, fibroblast-like cells in G47 (passage 19). Phase-contrast, scale bar represents 50 μ.

ploidy was found in 3/3 grade IV and 1/2 grade III astrocytoma-derived cultures; a diploid peak was found in 1/2 grade III and 1/1 grade II astrocytoma-derived culture.

*Chromosome analysis* was successfully carried out on four cell lines, G47, G223, G226 and G233 (Table I, last column). G47 had a wide range of numerical and structural changes. A total of 15 metaphases was karyotyped and the chromosome number varied between 74–79 and 126–155. There was no definite modal number. Clonal structural changes involved chromosomes 1, 5, 6, 7, 9, 11, 17 and 19. A representative karyotype is shown in Figure 2. Two copies of the structurally abnormal chromosomes were present in the cells with 126–155 chromosomes, demonstrating that polyploidisation had occurred during tumour progression. Cell lines G223 and G233 were in the diploid range. G223 had a complex hypoploid mainline karyotype with structural variation involving chromosomes 1, 2, 3, 4, 5, 6, 7, 9 and 14. The Y chromosome and a chromosome 13 were missing. Cytogenetic studies were carried out twice on the cell line G233. In culture G233/a, analysis of eight metaphases revealed two cells with a translocation t(6;8)(p12;p22). Analysis of a parallel culture (G233/b) demonstrated the presence of an abnormal cell line with the mainline karyotype 48,XX,+3,+7,add(13)(p11). Cell line G226 had an apparently normal karyotype.

*Effects of histamine and histamine antagonists on proliferation*

Cells were cultured at suboptimal conditions in order to be able to measure positive and negative effects of the added factors on proliferation. In most cultures this was realised using 1% serum. However, cell line U138MG and U373MG were tested at 0.5% and 0.25% serum, respectively, because of high proliferation rate at 1% serum. Figure 3a shows an example of the growth curves in one such an experiment. The cell line G15 showed maximal proliferation with 10% FCS, giving a population doubling time of 44 h in the middle log phase, which is transported to Table I. The effects of histamine, cimetidine and pyrilamine were measured during logarithmic growth (day 3–10) in 1% FCS. The differences in number of population doublings between the growth curves and the control growth curve (1% FCS) were calculated and transported to Figure 3b and Table II.

In this table, the results of the stimulation/inhibition experiments are summarised. Cell lines G233/a and G233/b are considered as separate cultures. The addition of 0.2 mM histamine induced significant stimulation of proliferation in primary cell lines both tested with 1% FCS (3/9 cases) or 1% AB serum (2/3 cases), and also in continuous cell lines (2/4 cases tested with FCS and in 1/4 tested with AB serum). Inhibition of proliferation was found in one case (U87MG). Addition of 0.2 μM histamine induced significant stimulation of proliferation in 0/2 continuous cell lines and in 2/2 primary cell lines, but only when tested with AB serum. Without serum addition, 0.2 μM histamine induced significant stimulation in 2/3 primary and in 1/1 continuous cell lines. In summary, proliferation of 2/4 continuous cell lines and of 9/13 primary cell lines was stimulated with histamine in one or the other tested condition, inhibition occurred in 1/4 continuous cell lines.

The addition of 1 mM cimetidine induced significant inhibition of proliferation in 2/4 continous cell lines when tested with FCS and in 3/4 cases lines when tested with AB serum. The proliferation of the primary cell lines was inhibited significantly with 1 mM cimetidine in 4/13 cases when tested with FCS and in 4/5 cases when tested with AB serum, making a total of 6/13 primary cell lines that were reactive to cimetidine.

The effect of the addition of 0.1 mM pyrilamine was variable. The combined results of FCS and AB serum show that inhibition was found in 3/4 continuous cell lines and in 2/8 primary cell lines, while 3/8 primary cell lines were stimulated.

Because of the consistent inhibiting effect of cimetidine, we tested the effect of lower concentrations in two responsive continuous cell lines and two responsive primary cell lines. Figure 4 shows that maximal inhibition is obtained at 1 mM cimetidine in all four cases and that there was a dose-response relation in two cell lines (U138MG and G226). G15 showed significant inhibition even at 1 μM cimetidine.

**Figure 2** Representative karyotypes of G47 (**a**) and G223 (**b**) (see Table I), arrows indicate clonal structural chromosome abnormalities.

**Figure 3** **a**, Growth curves of G15, illustrating the maximal observed growth rate at 10% FCS (— —) and the effect of histamine and histamine antagonists at 1% FCS. —— 1% FCS, - - - 0.2 mM histamine, - · - 1 mM cimetidine, --- 0.1 mM pyrilamine. **b** Changes in number of population doublings from day 3–10 due to histamine and histamine antagonists in the presence of 1% FCS, calculated from the growth curves in Figure 3a. ▩ histamine, ■ cimetidine, ▩ pyrilamine. *$P<0.05$, **$P<0.005$.

**Figure 4** Response of four glioma cell lines to different cimetidine doses, expressed as the difference between the number of population doublings of the control growth curve (1% FCS alone) and the test growth curve (1% FCS + cimetidine). The used concentrations were: ■ 1 mM, ▩ 0.1 mM, ▨ 10 μM, and ▨ 1 μM cimetidine. *$P<0.05$, **$P<0.005$ and ***$P<0.0005$.

### *Specificity of the measured inhibition*

In order to test the specificity of the observed inhibition with cimetidine and pyrilamine, the effect of addition of 0.2 mM histamine to the cimetidine-inhibited culture was tested in one continuous cell line (U138MG) and in two primary cell lines (G15 and G611). The same test was performed in the pyrilamine-inhibited culture of one continuous cell line (U138MG) and one primary cell line (G47). Figure 5a shows that histamine addition significantly reversed the cimetidine-induced effect in U138MG and in G611. Figure 5b shows that pyrilamine-induced inhibition was significantly reduced in both cases.

### *Correlation of cimetidine-induced growth inhibition with the maximal growth rate*

The observations with the primary cell lines suggested an inverse correlation between cimetidine responsiveness and the maximal growth rate of a given cell line, observed with 10% FCS addition. This was confirmed by calculating the linear regression between these two parameters (Figure 6). The correlation coefficient was − 0.73 with a chance probability

**Table II** Effects of histamine, cimetidine and pyrilamine on proliferation of glioma cell cultures

| | *FCS*[a] *his H*[d] | *hisL*[e] | *AB*[b] *hisH* | *hisL* | *O*[c] *hisL* | *FCS cim*[f] | *AB cim* | *FCS pyr*[g] | *AB pyr* |
|---|---|---|---|---|---|---|---|---|---|
| *Continuous cell lines* | | | | | | | | | |
| U87MG | ↓[h] | | ↓↓ | | ↑↑ | = | = | ↓↓ | = |
| U138MG | ↑↑ | = | ↑ | = | | ↓↓↓ | ↓↓↓ | ↓↓↓ | ↓↓↓ |
| U343MGa | = | | = | | | = | ↓↓ | = | = |
| U373MG | ↑↑ | = | = | = | | ↓↓ | ↓↓ | ↓↓↓ | ↓↓↓ |
| *Primary cell lines* | | | | | | | | | |
| PU-G15 | = | | ↑ | | | ↓↓↓ | ↓↓↓ | ↓↓↓ | = |
| PU-G24 | = | | ↑↑↑ | | | ↓↓↓ | ↓↓↓ | = | ↑↑↑ |
| PU-G47 | ↑ | | | | | ↓↓ | | ↓↓↓ | |
| PU-G162 | = | | = | | | = | ↓↓↓ | | = |
| PU-G177 | ↑ | | | | | = | | ↑↑ | |
| PU-G189 | ↑ | | | | | = | | = | |
| PU-G223 | = | | | | | = | | | |
| PU-G226 | | | | | ↑↑ | ↓↓ | | | |
| PU-G233/a | = | | | | ↑↑ | = | | = | |
| PU-G233/b | = | | | | = | = | | ↑↑ | |
| PU-G283 | | | | | | = | | | |
| PU-G311 | | = | | ↑↑ | | = | ↓↓↓ | | |
| PU-G611 | | = | | ↑↑↑ | | = | = | | |

[a]FCS: tested with foetal calf serum; [b]AB: tested with pooled human AB serum; [c]0: tested without serum addition; [d]hisH: 0.2 mM histamine; [e]hisL: 0.2 μM histamine; [f]cim: 1 mM cimetidine; [g]pyr: 0.1 mM pyrilamine; [h]stimulation or inhibition of cell proliferation: ↑/↓ <0.5 population doublings, ↑↑/↓↓ 0.5–1 population doublings, ↑↑↑/↓↓↓ >1 population doublings, = no stimulation or inhibition (scores represent the mean of 4–6 duplicates and were significant in the Student's '*t*' test). Serum was added in a concentration of 1%, except in U138MG and U373MG, which were tested at 0.5% and 0.25% serum, respectively.

**Figure 5** Effect of the addition of histamine on cimetidine-induced growth inhibition (**a**) and on pyrilamine-induced growth inhibition (**b**). The bars represent the difference between the number of population doublins of the control growth cruve (1% FCS alone) and the curve of the test. The used concentrations were: ▨ 1 mM cimetidine in **a**, 0.1 mM pyrilamine in **b**, ▩ the antagonist + 0.2 mM histamine. *$P<0.05$, **$P<0.005$ and ***$P<0.0005$.

of 0.011. No significant correlation was found for histamine-induced stimulation or pyrilamine-induced effects.

### Discussion

### *Characterisation*

Positive staining for GFAP, found in 7/8 of our primary cultures, is indicative for a glial phenotype. Low staining intensity in 1/8 of our cultures agrees with the finding of low GFAP contents reported for 1/5 continuous glioma cell lines (Ito *et al.*, 1989).

Cytogenic studies on cell lines G47 and G223 revealed complex karyotypes with structural and numerical variation. Both of these lines were derived from high grade tumours. The association between complex karyotypic changes and high malignancy in brain tumours has been reported recently by Kimmel *et al.* (1992). Of particular note is the presence of structural abnormalities involving the short arm of chromosome 9 in cell lines G47 and G223. Kimmel *et al.* (1992) also demonstrated that aberrations of 9p appear to be continued to brain tumours of high malignancy. Molecular studies using a range of probes for this region of chromosome 9 have shown that loss of DNA sequences occurs at a significant frequency in gliomas and may represent an important step in the progression of these tumours (Olufunmilayo *et al.*, 1992). Loss of heterozygosity for genetic loci on the long arm of chromosome 10 also occurs frequently in gliomas (Rasheed *et al.*, 1992); cell line G47 had a deletion of region 10q12q24. The other chromosomal abnormalities found in these two cell lines are typical of these reported for glioma (Jenkins *et al.*, 1989; Ransom *et al.*, 1992). Cytogenic studies were carried out twice on cell line G233. Line G233/a had been maintained serially in tissue culture from the original tumour biopsy. Line G233/b was derived from cells cultured after storage in liquid nitrogen at a very early stage of growth of the original tumour. In both lines G233/a and G233/b abnormal clones were identified, but with different chromosomal abnormalities within the diploid range. This suggests either that the observed karyotype changes are a culturing artefact or that they represent a selection of the original tumour cell population. Line G233/b had trisomy 7 which had been reported as a culturing artefact in normal brain cells (Heim *et al.*, 1989), but is also one of the more common changes found in gliomas (Bigner *et al.*, 1988; Arnoldus *et al.*, 1991). The normal karyotypes of culture G226 and the main population of culture G233/a may indicate that these cultures originated from gliomas with a comparatively homogeneous population of near-diploid cells, which form the majority of gliomas (Bigner & Mark, 1984).

**Figure 6** Regression curve representing the correlation between cimetidine-induced inhibition observed at 1% FCS and the maximal measured growth rate of primary glioma cell lines at 10% FCS; the cimetidine-induced inhibition is expressed as the difference between the number of population doublings between the control growth curve (serum alone) and the test curve (cimetidine added).

The finding with flow cytometry of an aneuploid peak in all four tested high-grade gliomas and diploid peak in one high-grade and one low-grade glioma agrees with the distribution found by others (Onda *et al.*, 1988; Spaar & Spaar, 1990).

Morphology and GFAP staining indicate that the used method was successul in establishing primary glioma cell cultures. As mentioned above, karyotyping and flow cytometry suggest a distribution and specificity of anomalies corresponding with those found for gliomas. Another argument for the glioma nature of our cultures is that the results of the continuous cell lines parallel those of the primary cell lines. On the other hand, it should be considered that it is most likely that even the early passage populations represent a selection of the original tumour heterogeneity (Shapiro *et al.*, 1981). In addition the findings with cytogenetic and flow cytometric studies in the near-diploid range may be of non-tumorous origin, and aneuploid findings may be generated *in vitro*, as we did not make direct preparations in order to identify original tumour characteristics (Shapiro *et al.*, 1981; Onda *et al.*, 1988).

### *Sensitivity to histamine and histamine antagonists*

We could detect significant histamine-induced stimulation of proliferation in 12/16 tested glioma cell lines. This effect was found both in the continuous and in the primary cell lines. The differences that were found under the various test conditions (FCS, AB-serum, no serum) reflect differences in proliferation modulating capacity of the sera used. A growth stimulating effect of histamine has been implicated in other *in vitro* systems. Stimulatory activity via $H_1$-receptors was found to occur in HeLa and A431 human carcinoma cells and A875 human melanoma cells (Tilly *et al.*, 1990); and in normal human lung fibroblasts (Jordana *et al.*, 1988), normal canine airway smooth muscle cells (Panetteiri *et al.*, 1990), normal rat astrocytes (Rodriguez *et al.*, 1989) and normal murine haematopoietic stem cells via the $H_2$-receptors (Schneider *et al.*, 1990).

Cimetidine significantly inhibits cell proliferation in the majority of cell lines in the present study. This indicates the dependence of proliferation of these cell lines on stimulation of the $H_2$-receptor. In three cell lines that showed no response to cimetidine in the presence of FCS, a significant inhibition was found in the presence of AB serum. This confirms the relative importance of histamine stimulation for cells cultured with AB serum.

At low serum concentrations pyrilamine induced significant inhibition in 5/11 tested cell lines and stimulation in 2/11 cell lines. Inhibition of cell proliferation was also found in HN-1 human squamous carcinomas cells (Bijman *et al.*, 1987) and normal human skin fibroblasts via $H_1$-receptors (Johnson & Johnson, 1990).

Three observations indicated that histamine antagonists induced a specific inhibition of proliferation and no non-specific cytotoxic effect. First, there was a cell line specific sensitivity to the addition of cimetidine or pyrilamine as these factors could not inhibit proliferation in all cell lines. Second, the cimetidine-induced inhibition of proliferation obtained at low FCS concentration was inversely correlated with the maximal growth rate observed at 10% FCS (Figure 6), suggesting a decreasing importance of stimulation of the $H_2$-receptor with increasing growth rate. Fast growing cell lines may have accumulated more mutations that make them sensitive to multiple growth stimuli (Den Otter *et al.*, 1990) and thereby less depending on each separate stimulus, *in casu* histamine. Third, the observed histamine antagonist-induced inhibitions were reversed in some cell lines. The effects of histamine and its antagonists were not related to histology or classification of the original tumour.

*Importance of histamine and histamine antagonists*

In five cell lines, addition of histamine in a concentration approaching the whole blood level (0.2 $\mu$M) could stimulate proliferation. In two cell lines (U138MG and U373MG), addition of 0.2 mM histamine was more effective than 0.2 $\mu$M. In this respect it is noteworthy that within a tumour the histamine level can rise due to local production (Ahlström *et al.*, 1966; Bartholeyns & Fozard, 1985; Johnston, 1967; Kahlson *et al.*, 1963; Mackay *et al.*, 1960). This implicates that even *in vivo* cells with low sensitivity to histamine may be stimulated to proliferate by this factor. Another implication of the histamine-forming capacity of tumour cells is that autocrine stimulation of glioma cell proliferation may be involved. Therefore, the histamine antagonist-induced inhibition of proliferation may reflect interference with stimulation by nascent histamine rather than with stimulation by serum-derived histamine.

Our experiments indicate that both $H_2$- and $H_1$-receptors can be involved in the proliferation of glioma cells. Both receptors can be active on the same cell, as is illustrated by the histamine-induced vasodilatation, where both $H_1$- and $H_2$-receptors are active in the relaxation of smooth muscle cells of small vessels (Douglas, 1985). An alternative explanation is that in the glial tumours and the cultures that were derived from these tumours, two different cell populations were present, one bearing $H_1$-receptors and the other bearing $H_2$-receptors. In a cultured glial tumour several subpopulations can be present (Westermark *et al.*, 1985; Shapiro *et al.*, 1981).

Cimetidine-induced inhibition was most prominent at a high concentration, that would be toxic *in vivo*. However, in the physiological situation, cell proliferation is regulated by the concerted action of balanced concentrations of various growth factors with stimulating and inhibiting actions (Sporn & Roberts, 1985; 1988). The *in vitro* system lacks this delicate balance of stimuli. Therefore the impact of a lower concentration of a histamine receptor-blocking agent may be more prominent *in vivo*. This is supported by the obtained *in vivo* tumour growth-inhibiting effect of non-toxic concentrations of $H_2$-receptor antagonists as mentioned in the Introduction. A particular problem concerning *in vivo* application of a water-soluble drug like cimetidine in the brain is the blood-brain barrier. However, this barrier has been reported to be non-functional in nearly all tumours (Wolff & Boker, 1989), or at least to a high extent in different gliomas or in different regions of a tumour (Shapiro & Shapiro, 1986). This is due to ultrastructural changes like interendothelial junction abnormalities and fenestrations in tumour vessel walls, leading to increased permeability (Stewart *et al.*, 1985).

As noticed, subculturing human gliomas induces selection. Nevertheless, the high proportion of cultures that was reactive to histamine and its antagonists suggests a widespread sensitivity.

### Conclusions

We conclude that histamine can be a potent growth stimulating factor for cell cultures that originate from human glial tumours. The $H_2$-receptor was involved more consistently in this growth stimulation than the $H_1$-receptor, and blocking the $H_2$-receptor induced growth inhibition in high proportion of the cultures. *In vivo*, histamine $H_2$-receptor antagonists can prolong survival in hosts bearing various human and animal tumours. Therefore, $H_2$-receptor antagonists are potential candidates for the postsurgical treatment of glioma patients, depending on the extent of breakdown of the blood-brain barrier in the tumour.

We thank Prof Dr C.A.F. Tulleken and the staff of the Department of Neurosurgery of the Academical Hospital Utrecht for their co-operation in selecting appropriate material. We are grateful to Annie Oostveen and the personnel of the operation room for their dedicated attention in the direct postsurgical care of the removed tissues. We thank the Maurits and Anna De Kock Stichting for contributory laboratory equipment.

### References

AHLSTROM, C.G., JOHNSTON, M. & KAHLSON, G. (1966). Histamine formation in tumor-bearing rats. *Life Sci.*, **5**, 1633.

ARMITAGE, J.O. & SIDNER, R.D. (1979). Antitumour effect of cimetidine? *Lancet*, **i**, 882.

ARNOLDUS, E.P.J., NOORDERMEER, J.A., PETERS, A.C.B., VOORMOLEN, J.H.C., BOTS, G.T.A.M., RAAP, A.K. & VAN DER PLOEG, M. (1991). Interphase cytogenetics of brain tumours. *Genes. Chromosomes & Cancer*, **3**, 101.

BARTHOLEYNS, J. & FOZARD, J.R. (1985). Role of histamine in tumor development. *Trends Pharmacol. Sci.*, **7**, 123.

BIGNER, S.H. & MARK, J. (1984). Chromosome and chromosomal progression of human gliomas *in vivo*, *in vitro* and in athymic nude mice. *Prog. Exp. Tumor. Res.*, **27**, 67.

BIGNER, S.H., MARK, J., BURGER, P.C., MAHALEY, M.S. Jr, BULLARD, D.E., MUHLBAIER, L.H. & BIGNER, D.D. (1988). Specific chromosomal abnormalities in malignant human gliomas. *Cancer Res.*, **48**, 405.

BIJMAN, J.T., WAGENER, D.J., GRAAFSMA, S.J., WESSELS, J.M. & VAN DEN BROEK, P. (1987). Modulation of proliferation of a human head and neck squamous carcinoma cell line (HN-1) by catecholamines and histamine. *Anticancer Res.*, **7**, 147.

BURTIN, C., NOIROT, C., SCHEINMANN, P., GALOPPIN, L., SABOLOVIC, D. & BERNARD, P. (1988). Clinical improvement in advanced cancer disease after treatment combining histamine and H2-antihistaminics (ranitidine or cimetidine). *Eur. J. Cancer Clin. Oncol.*, **24**, 161.

BURTIN, C., SCHEINMANN, P., SALOMON, J.C., LESPINATS, G. & CANU, P. (1982). Decrease in tumour growth by injections of histamine or serotonin in fibrosarcoma-bearing mice: influence of $H_1$ and $H_2$ histamine receptors. *Br. J. Cancer*, **45**, 54.

DAUMAS-DUPORT, C., SCHEITHAUER, B., O'FALLON, J. & KELLY, P. (1988). Grading of astrocytomas, a simple and reproducible method. *Cancer*, **62**, 2152.

DEN OTTER, W., KOTEN, J.W., VAN DER VEGT, B.J.H., BEEMER, F.A., BOXMA, O.J., DERKINDEREN, D.J., DE GRAAF, P.W., HUBER, J., LIPS, C.J.M., ROHOLL, P.J.M., SLUIJTER, F.J.H., TAN, K.E.W.P., VAN DER HEYDEN, K.A., VAN DER VEN, L.T.M., & VAN UNNIK, J.A.M. (1990). Oncogenesis by mutations in anti-oncogenes: a view. *Anticancer Res.*, **10**, 475.

DOUGLAS, W.W. (1985). Histamine and 5-hydroxytryptamine (serotonin) and their antagonists. In Goodman Gilman, A., Goodman, L.S., Kall, T.W. & Murad, F. (eds.), *The Pharmacological Basis of Therapeutics*. Macmillan Inc., New York, p. 609.

FORSBY, N., COLLINS, V.P., BRUNK, U.T., FREDRIKSON, B.A. & WESTERMARK, B. (1986). Translocation of human glial and glioma cells in culture. *Virchows Arch. B*, **51,** 3.

FRESHNEY, R.I. (1980). Tissue culture of glioma of the brain. In Thomas, D.G.T. & Graham, D.I. (eds.), *Brain Tumours-Scientific Basis, Clinical Investigation and Current Therapy*. Butterworths, London, p.21.

HEIM, S., MANDAHL, N., JIN, Y., STROMBLAD, S., LINSTROM, E., SALFORD, L.G. & MITELMAN, F. (1989). Trisomy 7 and sex chromosome loss in human brain tissue. *Cytogenet. Cell Genet.*, **52,** 136.

HSU, S.-M., ROHOLL, P.J.M., KO, Y.H. & LOK, M.-S. (1989). Malignant fibrous histiocytoma is not related to histiocytes: consistent phenotypic expression of CD13, CD10, and vimentin in four malignant fibrous histiocytoma cell lines. *Cancer J.*, **3,** 423.

ITO, M., NAGASHIMA, T. & HOSHINO, T. (1989). Quantitation and distribution analysis of glial fibrillary acidic protein in human glioma cells in culture. *J. Neuropathol. Exp. Neurol.*, **48,** 560.

JENKINS, R.B., KIMMEL, D.W. MOERTEL, C.A., SCHULTZ, C.G., SCHEITHAUER, B.W., KELLY, P.J. & DEWALD, G.W. (1989). A cytogenic study of 53 human gliomas. *Cancer Genet. Cytogenet.*, **39,** 352.

JOHNSON, C.L. & JOHNSON, C.G. (1990). Inhibition of human skin fibroblast proliferation by histamine and phorbol esters is mediated by protein kinase C. *Cell. Signal.*, **2,** 105.

JOHNSTON, M. (1967). Histamine formation in rats bearing the Walker mammary carcinosarcomas. *Experientia*, **23,** 152.

JORDANA, M., BEFUS, A.D., NEWHOUSE, M.T. BIENENSTOCK, J. & GUALDIE, J. (1988). Effect of histamine on proliferation of normal human adult lung fibroblasts. *Thorax*, **43,** 552.

KAHLSON, G., ROSENGREN, E. & STEINHARDT, D. (1963). Histamine forming capacity of multiplying cells. *J. Physiol.*, **169,** 487.

KAHLSON, G. & ROSENGREN, E. (1968). New approaches to the physiology of histamine. *Physiol. Rev.*, **48,** 155.

KERNOHAN, J.W., MABON, R.F., SVIEN, H.J. & ADSON, A.W. (1949). Symposium on a new and simplified concept of gliomas. A simplified classification of gliomas. *Proc. Mayo Clin.*, **24,** 71.

KIMMEL, D.W., O'FALLON, J.R., SCHEITHAUER, B.W., KELLY, P.J., DEWALD, G.W. & JENKINS, R.B. (1992). Prognostic value of cytogenic analysis in human cerebral astrocytomas. *Ann. Neurol.*, **31,** 534.

KORNBLITH, P.L. & WALKER, M. (1988). Chemotherapy for malignant gliomas. *J. Neurosurg.*, **68,** 1.

LEIBEL, S.A. & SHELINE, G.E. (1987). Radiation therapy for neoplasms of the brain. *J. Neurosurg.*, **66,** 1.

MACKAY, D., MARSHALL, P.B. & RILEY, J.F. (1960). Histidine decarboxylase activity in a malignant rat hepatoma. *J. Physiol.*, **153,** 31p.

NAZARRO, J.M. & NEUWELT, E.A. (1990). The role of surgery in the management of supratentorial intermediate and high-grade astrocytomas in adults. *J. Neurosurg.*, **73,** 331.

NISTÉR, M., HELDIN, C.-H. & WESTERMARK, B. (1986). Clonal variation in the production of a platelet-derived growth factor-like protein and expression of corresponding receptors in human malignant glioma. *Cancer Res.*, **46,** 332.

OLUFUNMILAYO, I.O., JENKINS, R.B., RANSOM, D.T., MALIK, K., POMYKALA, H., NOBORI, T., COWAN, J.M., ROWLEY, J.D. & DIAZ, M.O. (1992). Molecular analysis of deletions of the short arm of chromosome 9 in human gliomas. *Cancer Res.*, **52,** 2523.

ONDA, K., TANAKA, R., WASHIYAMA, K., TAKEDA, N. & KUMANISHI, T. (1988). Correlation of DNA ploidy and morphological features of human glioma cell cultures with the establishment of cell lines. *Acta Neuropathol. Berl.*, **76,** 433.

PANETTIERI, R.A., YADVISH, P.A., KELLY, A.M., RUBINSTEIN, N.A. & KOTLIKOFF, M.I. (1990). Histamine stimulates proliferation of airway smooth muscle and induces c-fos expression. *Am. J. Physiol.*, **259,** L365.

PELLETIER, B., DHAINAUT, F., PAULY, A. & ZAHND, J.P. (1988). Evaluation of growth rate in adhering cell cultures using a simple colorimetric method. *J. Biochem. Biophys. Methods*, **16,** 63.

PLEASURE, D., KREIDER, B., SOBUE, G., ROSS, A.H., KOPROWSKI, H., SONNENFELD, K.H. & RUBENSTEIN, A.E. (1986). Schwann-like cells cultured from human dermal neurofibromas. Immunohistological identification and response to Schwann cell mitogens. *Ann. N.Y. Acad. Sci.*, **486,** 227.

PONTÉN, J. & MACINTYRE, E.H. (1968). Long term culture of normal and neoplastic human glia. *Acta Pathol. Microbiol. Scand.*, **74,** 465.

RANSOM, D.T., RITLAND, S.R., MOERTAL, C.A., DAHL, R.J., O'FALLON, J.R., SCHEITHAUER, B.W., KIMMEL, D.W., KELLY, P.J., OLOPADE, O.I., DIAZ, M.O. & JENKINS, R.B. (1992). Correlation of cytogentic analysis and loss of heterozygosity studies in human diffuse astrocytomas and mixed oligo-astrocytomas. *Genes, Chromosomes & Cancer*, **5,** 357.

RASHEED, B.K.A., FULLER, G.N., FRIEDMAN, A.H., BIGNER, D.D. & BIGNER, S.H. (1992). Loss of heterozygosity for 10q loci in human gliomas. *Genes, Chromosomes and Cancer*, **5,** 75.

RODRIGUEZ, J., MORAN, J., BLANCO, I. & PATEL, A.J. (1989). Effect of histamine on the development of astroglial cells in culture. *Neurochem. Res.*, **14,** 693.

RUTGERS, D.H. (1981). Cell suspension preparation of solid tumours for flow cytometry. *Acta Pathol. Microbiol. Scand.*, (suppl). **274,** 67.

SCHEIDER, E., PIQUET PELLORCE, C. & DY, M. (1990). New role for histamine in interleukin-3-induced proliferation of hematopoietic stem cells. *J. Cell. Physiol.*, **143,** 337.

SCOTCHER, S., SIKOVA, K. & FREEDMAN, L. (1981). Gastric cancer and cimetidine: does delay in diagnosis matter? *Lancet*, **ii,** 630.

SHAPIRO, J.R., YUNG, W.A. & SHAPIRO, W.R. (1981). Isolation, karyotype and clonal growth of heterogeneous subpopulations of human malignant gliomas. *Cancer Res.*, **41,** 23.

SHAPIRO, W.R. & SHAPIRO, J.R. (1986). Principles of brain tumor chemotherapy. *Semin. Oncol.*, **13,** 56.

SMITH, M.T., LUDWIG, C.L., GODFREY, A.D. & ARMBRUSTMACHER, V.W. (1983). Grading of oligodendrogliomas. *Cancer*, **52,** 2107.

SPAAR, F.W. & SPAAR, U. (1990). DNA in human glioblastomas. A flow-fluorescence cytometrical examination of 96 tumors. *Neurosurg. Res.*, **13,** 123.

SPORN, M.B. & ROBERTS, A.B. (1985). Autocrine growth factors and cancer. *Nature*, **313,** 745.

SPORN, M.B. & ROBERTS, A.B. (1988). Peptide growth factors are multifunctional. *Nature*, **332,** 217.

STEWART, P.A., HAYAKAWA, K., HAYAKAWA, E., FARRELL, C.L. & DEL-MAESTRO, R.F. (1985). A quantitative study of blood-brain barrier permeability ultrastructure in a new rat glioma model. *Acta Neuropathol. Berl.*, **67,** 96.

SVIEN, H.J., MABON, R.F., KERNOHAN, J.W. & ADSON, A.W. (1949). Astrocytomas. *Proc. Mayo Clin.*, **25,** 54.

TILLY, B.C., TERTOOLEN, L.G., REMORIE, R., LADOUX, A., VERLAAN, I., DE LAAT, S.W. & MOOLENAAR, W.H. (1990). Histamine as a growth factor and chemoattractant for human carcinoma and melanoma cells: action through Ca2(+)-mobilizing $H_1$ receptors. *J. Cell. Biol.*, **110,** 1211.

TUTTON, P.J.M. & BARKLA, D.H. (1978). Stimulation of cell proliferation by histamine $H_2$-receptors in dimethylhydracine-induced adenocarcinomata. *Cell Biol. Int. Rep.*, **2,** 199.

TUTTON, P.J.M. & BARKLA, D.H. (1987). Biogenic amines as regulators of the proliferative activity of normal and neoplastic intestinal epithelial cells (review). *Anticancer Res.*, **7,** 1.

TUTTON, P.J.M. & STEEL, G.G. (1979). Influence of biogenic amines on the growth of xenografted human colorectal carcinomas. *Br. J. Cancer*, **40,** 743.

TØNNESEN, H., KNIGGE, U., BÜLOW, S., DAMM, P., FISCHERMAN, K., HESSELFELDT, P., HJORTRUP, A., PEDERSEN, I.K., PEDERSEN, V.M., SIEMSSEN, O.J. & CHRISTIANSEN, P.M. (1988). Effect of cimetidine on survival after gastric cancer. *Lancet*, **ii,** 990.

WESTERMARK, B., NISTER, M. & HELDIN, C.-H. (1985). Growth factors and oncogenes in human malignant glioma. *Neurol. Clin.*, **3,** 785.

WOLFF, M. & BOKER, D.K. (1989). Immunohistochemical demonstration of immunoglobulins and albumin in human brain tumours. *Clin. Neuropathol.*, **8,** 72.